# The Protective Effects of PSM-04 Against Beta Amyloid-Induced Neurotoxicity in Primary Cortical Neurons and an Animal Model of Alzheimer’s Disease

**DOI:** 10.3389/fphar.2019.00002

**Published:** 2019-01-24

**Authors:** Hyunjun Park, Shinwoo Kang, Eunjoo Nam, Yoo-Hun Suh, Keun-A Chang

**Affiliations:** ^1^Department of Health Sciences and Technology, Gachon Advanced Institute for Health Sciences and Technology (GAIHST), Gachon University, Incheon, South Korea; ^2^Neuroscience Research Institute, Gachon University, Incheon, South Korea; ^3^Department of Pharmacology, Gachon University of Medicine and Science, Incheon, South Korea

**Keywords:** Alzheimer’s disease, *Polygala tenuifolia* Willdenow, PSM-04, neuroprotection, 5xFAD mice

## Abstract

*Polygala tenuifolia* Willdenow is a herb known for its therapeutic effects in insomnia, depression, disorientation, and memory impairment. In Alzheimer’s disease (AD) animal model, there has been no report on the effects of memory and cognitive impairment. PSM-04, an extract from the root of *P. tenuifolia* Willdenow, was developed with improved bioabsorption. The present study aimed to investigate the neuroprotective effects of PSM-04 on AD and reveal the possible molecular mechanism. The neuroprotective effect of PSM-04 in primary cortical neurons treated with L-glutamate, oligomeric Aβ, or H_2_O_2_. PSM-04 exhibited significant neuroprotective effects against neurotoxicity induced by L-glutamate or oligomeric Aβ was studied. PSM-04 exhibited significant neuroprotective effects against neurotoxicity induced by L-glutamate or oligomeric Aβ. Oxidative stress induced by ROS was monitored using the DCF-DA assay, and apoptosis was assessed using the TUNEL assay in primary cortical neurons treated with H_2_O_2_ or oligomeric Aβ. PSM-04 also decreased oxidative stress induced by H_2_O_2_ and apoptotic cell death induced by oligomeric Aβ. We evaluated the therapeutic effect of PSM-04 in 5xFAD (Tg) mice, an animal model for AD. PSM-04 was orally administered to 4-month-old 5xFAD mice for 2 months. To confirm the degree of cognitive impairment, a novel object recognition task was performed. The treatment with PSM-04 significantly alleviated cognitive impairments in Tg mice. In addition, amyloid plaques and gliosis decreased significantly in the brains of PSM-04-administered Tg mice compared with Tg-vehicle mice. Furthermore, the administration of PSM-04 increased the superoxide dismutase-2 (SOD-2) protein level in hippocampal brain tissues. Our results indicated that PSM-04 showed therapeutic effects by alleviating cognitive impairment and decreasing amyloid plaque deposition in Tg mice. Therefore, PSM-04 was considered as a potential pharmacological agent for neuroprotective effects in neurodegenerative diseases, including AD.

## Introduction

Alzheimer’s disease (AD) is the most common neurodegenerative disease and constitutes approximately two-thirds of all cases of dementia (Reitz et al., 2011). AD is pathologically characterized by amyloid plaque deposition, intracellular neurofibrillary tangles, and cognitive function impairment (Tanzi and Bertram, 2005). Clinical features of AD include memory loss, altered memory, and cognitive impairment (Burns and Iliffe, 2009). The costs of caring for patients with AD are increasing annually, which imposes tremendous financial and social burden on the community and patients’ families. Clinically prescribed medicines alleviate symptoms of AD but cannot provide a fundamental treatment (Tan et al., 2014). During the last decade, all phase III clinical trials of promising candidate drugs, such as solanezumab and verubecestat, have failed due to no cognitive improvement and adverse effects.

In recent times, researches have been conducted to search for novel active extracts or components derived from various natural products which can be used for the treatment of brain diseases (Howes et al., 2003; Houghton and Howes, 2005). These natural products have proven effective with low side effects (Bent, 2008). *Polygala tenuifolia* Willdenow is one of the main components of Kai-Xin-San (KXS), a well-known traditional Chinese herbal decoction, which has been widely used to treat mental depression and memory loss in China (Yan et al., 2015). The roots of *P. tenuifolia* Willdenow, a natural oriental plant, have been used for memory improvement and for the treatment of insomnia, amnesia, depression, and palpitations with anxiety (Liu et al., 2010). BT-11 was extracted from the dried root of *P. tenuifolia* Willdenow by ethanol distillation (Park et al., 2002). Previous studies showed that BT-11 has neuroprotective effects as it improved scopolamine- and stress-induced amnesia in rats (Park et al., 2002; Shin et al., 2009b). BT-11 reportedly enhances cognitive functions, including memory, in the elderly (Lee et al., 2009; Shin et al., 2009a). Recently, *BT-11* is reportedly non-genotoxic at the appropriate dose (Shin et al., 2015). We have developed new *P. tenuifolia* Willdenow extract, PSM-04, for improving bioabsorption by removing stearic acid from BT-11.

In the present study, we investigated the neuroprotective effect of PSM-04 against neurotoxicity induced by L-glutamate, oligomeric Aβ, or H_2_O_2_ in primary cortical neurons. We also checked the improvement of cognitive dysfunction and pathological changes in 5xFAD transgenic mice, an animal model for AD.

## Materials and Methods

### Chemicals and Antibodies

PSM-04 was provided by Braintropia (Korea) for research purposes. BDNF was purchased from Peprotech (United States). Donepezil, 6E10 antibody, hexafluoroisopropanol (HFP), and vitamin E were purchased from Sigma Aldrich (St. Louis, MO, United States). Anti-BDNF antibody was purchased from Abcam (Cambridge, United Kingdom), and anti-Mn-SOD antibody was purchased from Merck Millipore (Darmstadt, Germany).

### Rat Primary Cortical Neuron Culture

**FIGURE 1:**
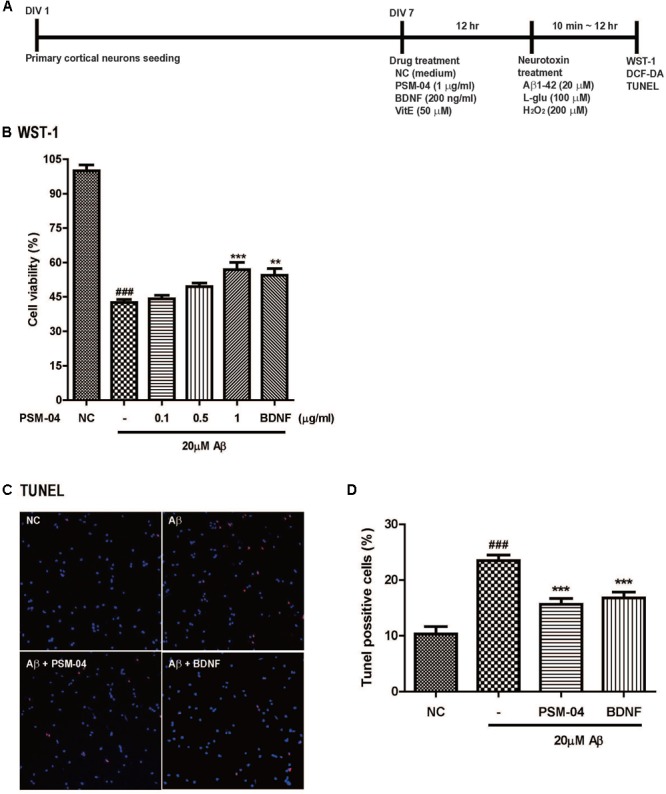
PSM-04 reduced the apoptotic cell death induced by oligomeric Aβ. **(A)** A schematic diagram of the *in vitro* experimental plan is shown. **(B–D)** Primary cortical neurons were pretreated with PSM-04 (0.1, 0.5, or 1 μg/mL) for 12 h and then treated with 20 μM of Aβ for 12 h. **(B)** Cell viability was determined by using WST-1 assay. All data are given as means ± standard error of the mean (SEM), and each experiment was repeated five times (*n* = 4–6 wells per group, *N* = 5). **(C)** The apoptotic cell death in primary cortical neurons was visualized by TUNEL staining (red) and counterstaining with DAPI (blue); consequently, 1-μg/mL PSM-04 or 200-ng/mL BDNF reduced the apoptotic cell death induced by 20 μM of Aβ. **(D)** The population of TUNEL-positive cells (red) is shown as a percentage compared to the total cell number. All data are given as means ± SEM and each experiment was repeated five times (*n* = 3 wells per group, *N* = 5). The statistical analyses were performed by one-way ANOVA followed by the Newman–Keuls *post hoc* test ^###^*p* < 0.001 vs. control (NC). ^∗^*p* < 0.05, ^∗∗^*p* < 0.01, and ^∗∗∗^*p* < 0.001 vs. Aβ only.

Pregnant Sprague–Dawley (SD) rats were purchased from Koatech (Korea). The cerebral cortex was dissected from an embryonic day 17 (E17) SD rat embryo and dissociated with a trypsin solution. The isolated cells (3 × 10^3^ cells) were plated on a 12-mm coverslip or a 96-well plate coated with poly-L-lysine (Sigma, United States). Primary cortical neurons were grown in Neurobasal medium supplemented with 2% B27, 2-mM L-glutamine, and 1% penicillin–streptomycin–amphotericin B mixture (Gibco BRL). Cultured media were changed every 2–3 days. Cortical neurons were cultured in a 5% CO_2_ humidified incubator at 37°C for 14–15 days. The *in vitro* experimental scheme is shown in Figure 1A. This study was performed in agreement with the principles of the Basel Declaration and recommendations of the Institutional Animal Care and Use Committee of the Lee Gil Ya Cancer and Diabetes Institute, Gachon University. The protocol was approved by the Institutional Animal Care and Use Committee of the Lee Gil Ya Cancer and Diabetes Institute, Gachon University (LCDI-2016-0061).

### Oligomeric Aβ Preparation

An oligomeric form of the Aβ_1-42_ peptide was prepared as described previously (Dahlgren et al., 2002). Vials containing 1 mg of the Aβ_1-42_-HFP film were allowed to thaw at room temperature for 10 min. Then, Aβ_1-42_ was dissolved in HFP at a concentration of 1 mmol/L. The HFP was removed under vacuum, and Aβ_1-42_ peptide film was stored at -20°C. The Aβ_1-42_ peptide film was dissolved in DMSO (Dochefa, Netherlands), and the peptide was then kept at -20°C in aliquots until use. For supporting oligomeric conditions, F-12 medium was added to the Aβ_1-42_ peptide and was incubated at 4°C for 24 h to oligomerize.

### Cell Viability Assay

To measure the metabolic activity of viable cells, WST-1 (Roche, Switzerland) or CCK-8 (Dojindo, Japan) assay was performed according to the manufacturer’s instructions. Primary cortical neurons were plated at a density of 3 × 10^3^ cells/well in the 96-well plate. Next, 1 μg/mL PSM-04 or medium was added into the media for pretreatment of primary cortical neurons (DIV 7-8) 12 h before treatment with 20 μM of the oligomeric form of Aβ_1-42_ or F12 medium. Thereafter, 200 ng/mL BDNF was used as positive control. Then, WST-1 or CCK-8 reagent was added to each well, and primary cortical neurons were additionally incubated at 37°C in 5% CO_2_ for 2 h. The absorbance of control or treated samples was measured using a multi-label plate reader (PerkinElmer, VICTOR X4) at OD of 450 nm.

### Measurement of ROS Generation

Cellular ROS formation was evaluated in primary cortical neurons using the DCF-DA assay kit (Abcam, United States) or DCF-DA compound (Sigma, United States). Briefly, primary cortical neurons were plated at a density of 3 × 10^3^ cells/well in 96-well optical black plates. PSM-04 was added into the medium for the pretreatment of primary cortical neurons (DIV8) for 12 h. The cultured medium was removed and replaced with 5-μM DCF-DA-treated Hank’s balanced salt solution (HBSS) for 20 min in the dark at 37°C. Then, cortical neurons were treated with 20-μM H_2_O_2_ and incubated for 10 min. BDNF (200 ng/mL) was used as positive control. The fluorescence was detected using a multi-label plate reader with excitation wavelength of 485 nm and emission wavelength of 535 nm.

### Measurement of Apoptotic Cell Death With the TUNEL Assay

The terminal deoxynucleotidyl transferase dUTP nick end labeling (TUNEL) staining kit was purchased from Roche (Switzerland). Apoptotic cell death was measured using the TUNEL assay following the manufacturer’s protocol. Briefly, primary cortical neurons were plated on prepared 12-mm coverslips, and 1-μg/mL PSM-04 was added into the media for the pretreatment of primary cortical neurons (DIV7) at 12 h before treatment with 20-μM Aβ_1-42_ or medium. BDNF (200 ng/mL) was used as positive control. Continually, primary cortical neurons were fixed with 4% paraformaldehyde in 4% sucrose and were then permeabilized for 2 min on ice. Primary cortical neurons were treated with the TUNEL reaction mixture and incubated for 60 min at 37°C. The nucleus was counterstained with DAPI. The nuclei were stained with DAPI and identified as a control. At the end of this procedure, coverslips were mounted on a slide glass and visualized by confocal microscopy (Olympus Laser scanning microscope, Japan). TUNEL-positive cells were counted by Image J.

### Animals and PSM-04 Treatment

5xFAD transgenic (Tg) mice expressing five human APP and PS1 genes [three in APP (Swedish mutation: K670N, M671L; Florida mutation: I716V; London mutation: V717I) and two in PS1 (M146L, L286V)] were donated by Seoul National University and maintained by crossing hemizygous transgenic mice with B6SJL F1 mice. A 12L:12D photoperiod was provided, and the temperature and humidity of the breeding room were automatically maintained at 22°C ± 2°C and 50 ± 10%, respectively. Food and water were provided *ad libitum* during the acclimation period to the polycarbonate cage.

PSM-04 or vehicle (saline) was orally administered to 4-month-old Tg or WT mice for 2 months, followed by the experimental scheme in Figure 3A. Each experimental group comprised 6–10 male mice per the following group: *vehicle*-treated *wild-type mice* (WT-v, *n* = 10); 5-mg/kg PSM-04-treated *wild-type mice* (WT-PP-5, *n* = 10); *vehicle*-treated Tg *mice* (Tg-v, *n* = 8); 5-mg/kg PSM-04-treated Tg *mice* (Tg-PP-5, *n* = 8); 10-mg/kg PSM-04-treated Tg *mice* (Tg-PP-10, *n* = 8); and donepezil-treated Tg *mice* (Tg-DP, *n* = 6) (Figure 3A). This study was performed in agreement with the principles of the Basel Declaration and recommendations of the Institutional Animal Care and Use Committee of the Lee Gil Ya Cancer and Diabetes Institute, Gachon University. The protocol was approved by the Institutional Animal Care and Use Committee of the Lee Gil Ya Cancer and Diabetes Institute, Gachon University (LCDI-2015-0025).

### Novel Objective Recognition Task

We conducted Novel objective recognition (NOR) task with the six groups of Tg mice to assess the changes in cognition and memory. NOR task was performed as described previously (Leger et al., 2013). The setup comprised a black-walled square box measuring 40 × 40 × 40 cm^3^. On the first day, mice were placed in the middle of the open-field box and allowed to adapt for 30 min. Next day, two same objects were placed in the box, and mice were habituated for 30 min. Last day, one object was changed to a novel object, and the exploration time of novel or familiar object was recorded for 5 min using the EthoVision XT 9 system (Noldus Information Technology, Wageningen, Netherlands). The following parameters were measured: the total exploration time, frequency, objective recognition time, and the memory index. The memory index is calculated by the exploration time for each object divided by the total exploration time.

### Immunohistochemistry

The mice were anesthetized with Zoletil and Rompun mixture (1 mg/g, ip) and euthanized by transcardial perfused with saline. Brain hemisphere tissues were fixed in 4% paraformaldehyde at 4°C for 24 h and were then dehydrated and paraffin-embedded. Paraffin-embedded tissues were cut at 4-μm thickness from the hippocampal region using a microtome (Thermo Electron Corporation, United States). Serial sections were placed on a slide glass. The brain slides were placed in a 60°C incubator for 1 h, rinsed with xylene for deparaffinization, and washed by ethanol series for dehydration. The brain slides were retrieved by treatment with 0.01-M citric acid (pH 6.0) for 10 min at 60°C and washed with 0.5% Triton X-100 in Tris-buffered solution. The slides were incubated with primary antibody (6E10) overnight at 4°C in Tris-buffered solution. For DAB staining, the tissue slide was incubated with liquid DAB substrate chromogen (DAKO, Japan) for 10 min at room temperature. The slides were washed with PBS and coverslipped with mounting solution. Extracellular Aβ load was evaluated in the cortex and the dentate gyrus of the hippocampus using a Zeiss AxioImager Z1 microscope equipped with Axiocam HRC camera and the Image J software (V1.4.3.67, NIH, United States). Serial images of 40 × or 100 × magnification were captured on an average of 2–3 sections per animal. The Aβ plaque load in the same brain region of the same size was measured with blind count and presented as numbers in the area.

### Western Blotting

For Western blot, brain tissues were lysed with RIPA buffer containing a cocktail of protease inhibitors (Roche Science, Mannheim, Germany) and a cocktail of phosphatase inhibitors (Sigma Aldrich). Protein was loaded on 8%–12% SDS–PAGE gel and transferred onto a PVDF membrane (Merck). Then, membranes were incubated in 6% skim milk for 1 h at room temperature. The primary antibody (SOD2 or β-actin) was incubated overnight at 4°C. After washing with TBS-T, membranes were incubated with the proper secondary antibody for 1 h at room temperature. The membranes were detected using the Pico EPD Western blot detection kit (ELPIS-Biotech, South Korea). The immunoblots were imaged using BLUE detection medical X-ray film (AGFA, Mortsel, Belgium). Quantification of blots was analyzed using the Image J software.

### Statistical Analysis

Statistical analysis was performed using the one-way analysis of variance (ANOVA) followed by the Newman–Keuls *post hoc* test. All data were expressed as mean ± standard error of the mean (SEM) value. The difference was considered statistically significant for ^∗^*p* < 0.05, ^∗∗^*p* < 0.01, and ^∗∗∗^*p* < 0.001. All calculations were performed using SPSS 23 (IBM, United States) or GraphPad Prism software (GraphPad Software Inc.).

## Results

### PSM-04 Did Not Induce Cytotoxicity in Primary Cortical Neurons

We first checked whether PSM-04 causes cytotoxicity in primary cortical neurons or not. To measure the cytotoxicity of PSM-04, we treated primary cortical neurons with various concentrations of PSM-04 (0.1, 0.5, 1, and 5 μg/mL) for 24 h and then evaluated the cell viabilities by a WST-1 assay. The treatment of PSM-04 did not induce cytotoxic events (Supplementary Figure S1A).

#### Neuroprotective Effect of PSM-04 Against the Excitotoxicity Induced by L-Glutamate Treatment

We studied the neuroprotective effects of PSM-04 against the neurotoxicity induced by L-glutamate. Glutamate plays an important role in neurotransmission in the brain, but it causes cell death if it is present in excess (Amor et al., 2010), leading to neurodegenerative diseases, such as AD. To measure the excitotoxic effects of L-glutamate, we evaluated cell viability by performing the CCK-8 assay after treatment of L-glutamate at various concentrations in primary cortical neurons for 2 h. Based on this result, we treated primary cortical neurons with 100-μM L-glutamate for 2 h after they were treated with 1-μg/mL PSM-04 or 200-ng/mL BDNF for 12 h. Then, we performed the CCK-8 assay (Supplementary Figure S2A) and TUNEL staining (Supplementary Figure S2B) to evaluate apoptotic cell death. The cell viability of the L-glutamate-treated cell decreased compared to that of the control (36.01 ± 1.62%) (Supplementary Figure S2A). PSM-04 rescued L-glutamate -induced cell death in a dose-dependent manner and showed a protective effect at 0.5 and 1 μg/mL (41.57 ± 1.47%, *p* < 0.001 and 42.88 ± 1.30%, *p* < 0.001, respectively) significantly; BDNF treatment also increased cell viability (48.99 ± 1.95%, *p* < 0.001) (Supplementary Figure S2A). In Supplementary Figure S2C, the 100-μM L-glutamate treatment (32.82% ± 1.26%) increased apoptosis compared with the control (16.64% ± 1.19%). Primary cortical neurons pre-treated with PSM-04 or BDNF showed that apoptotic cell death was reduced (20.2 ± 1.19%, *p* < 0.001; 20.13 ± 1.26%, *p* < 0.001) compared with the L-glutamate -only treatment (Supplementary Figure S2C).

#### Protective Effect of PSM-04 on the Neurotoxicity Induced by Oligomeric Aβ_1-42_

We checked the neuroprotective effects of PSM-04 against oligomeric Aβ_1-42_ peptide-induced neurotoxicity. Oligomeric Aβ_1-42_ peptides (Aβ) play a pathological role in neurodegenerative diseases in AD (Neves et al., 2008). After pretreatment with various concentrations of PSM-04 (0.1, 0.5, 1, and 5 μg/mL) for 12 h, 20 μM of Aβ was treated for 12 h, and the WST-1 assay was performed to confirm cell viability. In this condition, the cell viability of the Aβ-treated cell decreased compared to that of the control (42.63 ± 1.28%) (Figure 1B). PSM-04 rescued Aβ-induced cell death in a dose-dependent manner and showed a protective effect at 1 μg/mL (56.87 ± 3.24%, *p* < 0.001) significantly; BDNF treatment also increased cell viability (54.49 ± 2.93%, *p* < 0.001) (Figure 1B).

Next, TUNEL staining was performed to confirm that apoptotic cell death was reduced by PSM-04. Primary cortical neurons were treated with 1 μg/mL of PSM-04 for 12 h, and then, were treated with 20 μM of Aβ for 12 h. The apoptotic cell death was visualized by TUNEL staining (red) and counterstaining with DAPI (blue) (Figure 1C and Supplementary Figure 3). Primary cortical neurons pre-treated with PSM-04 showed that apoptotic cell death induced by Aβ was reduced (15.83 ± 1.22%, *p* < 0.001) compared to that observed with Aβ-only treatment (23.2 ± 1.18%); further, BDNF treatment also reduced apoptotic cell death (16.53 ± 1.35%, *p* < 0.001) (Figure 1D).

### Neuroprotective Effect of PSM-04 on the ROS Generation Induced by H_2_O_2_

We studied the neuroprotective effects of PSM-04 on the oxidative stress induced by H_2_O_2_. H_2_O_2_ induces oxidative stress through ROS production in neurodegenerative diseases (Uttara et al., 2009).

**FIGURE 2:**
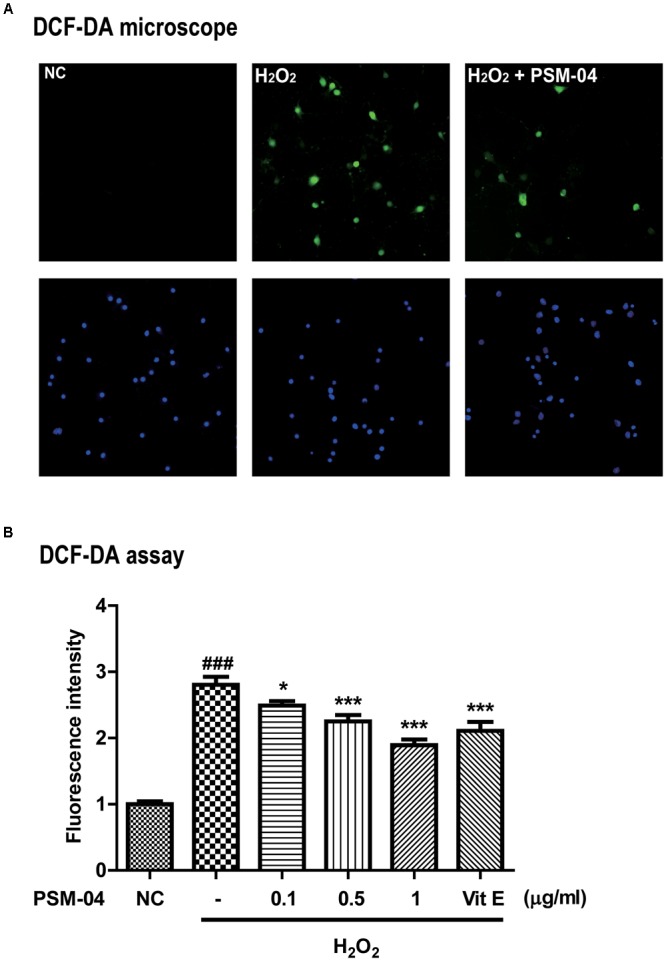
PSM-04 reduced the ROS generated by H_2_O_2_. Primary cortical neurons were treated with PSM-04 (0.1, 0.5, and 1 μg/mL) at 12 h before 200-μM H_2_O_2_ treatment. ROS production was determined by the DCF-DA assay. **(A)** Primary cortical neurons were stained with DCF-DA (green), counterstained with DAPI (blue), and visualized via fluorescence microscopy. It was seen that 1-μg/mL PSM-04 reduced the number of oxidative stress-induced cells by 200-μM H_2_O_2_ treatment. **(B)** The quantification of DCF-DA fluorescence was shown as a ratio versus the control. PSM-04 or vitamin E significantly reduced the fluorescence intensity (ROS production) induced by 200-μM H_2_O_2_ treatment. All data are given as means ± SEM, and each experiment was repeated five times (*n* = 4–6 wells per group, *N* = 5). The statistical analyses were performed by one-way ANOVA followed by the Newman–Keuls *post hoc* test. ^###^*p* < 0.001 vs. control (NC). ^∗^*p* < 0.05, ^∗∗^ *p* < 0.01, and ^∗∗∗^*p* < 0.001 vs. H_2_O_2_ only.

We checked that PSM-04 reduced the intracellular ROS produced by H_2_O_2_ treatment in primary cortical neurons. To measure the ROS induced by H_2_O_2_, we treated primary cortical neurons with various concentrations of H_2_O_2_ for 10 min and evaluated the ROS level by the DCF-DA assay. H_2_O_2_ treatment increased ROS levels in a dose-dependent manner (Supplementary Figure S1B). Subsequently, we treated primary cortical neurons with 200-μM H_2_O_2_ for 10 min after pretreatment with 0.1, 0.5, and 1-μg/mL PSM-04 for 12 h. In DCF-DA staining, enhanced oxidative stress was observed in primary cortical neurons treated with H_2_O_2_ via fluorescence microscopy (Figure 2A). The fluorescence intensity was expressed as a ratio to the untreated control (NC) group. Primary cortical neurons pretreated with PSM-04 (H_2_O_2_ + 0.1 μg/mL PSM-04, 2.49 ± 0.08, *p* < 0.05; H_2_O_2_ + 0.5-μg/mL PSM-04, 2.22 ± 0.12, *p* < 0.001; H_2_O_2_ + 1-μg/mL PSM-04, 1.80 ± 0.09, *p* < 0.001) showed reduced ROS generation compared to H_2_O_2_-only treatment (2.90 ± 0.14). In this result, H_2_O_2_-induced ROS production was significantly reduced by PSM-04 (Figure 2B).

These results indicated that PSM-04 increased cell viability and reduced apoptosis and ROS generation.

### PSM-04 Alleviated Cognitive Impairment in 5xFAD Mice

**FIGURE 3:**
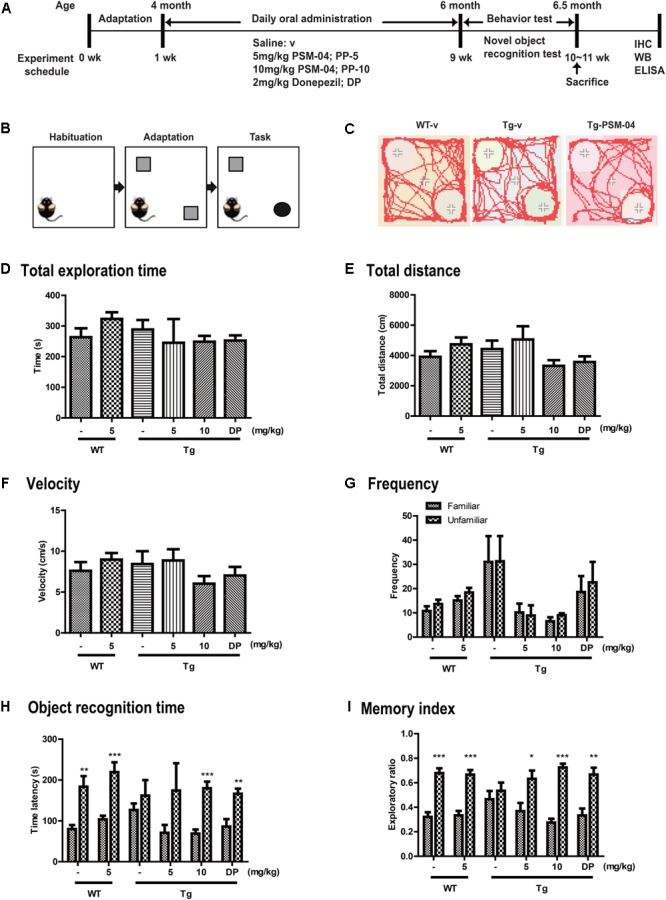
PSM-04 alleviated cognitive impairment in 5xFAD mice. **(A)** A schematic diagram of the *in vivo* experimental plan is shown. **(B)** The design of a novel object recognition (NOR) task is shown; in the habituation phase (30 min duration), mice were adapted to the open-field box. In the adaptation phase, the mice were exposed to two same objects. On the last day, one object was changed to a novel object and recorded for 10 min. **(C)** Representative track sheets showed alteration in locomotion and exploratory behavior in the NOR task (distance traveled to familiar vs. unfamiliar object). **(D–I)** Graphs represent total exploration time **(D)**, total distances **(E)**, velocity **(F)**, frequency **(G)**, objective recognition time **(H)**, and the memory index **(I)**. The statistical analyses were performed by one-way ANOVA, and data are presented as means ± SEM. ^∗^*p* < 0.05, ^∗∗^*p* < 0.01, and ^∗∗∗^*p* < 0.001. Vehicle-treated WT mice (WT-v, *n* = 8); 5-mg/kg PSM-04-treated WT mice (WT-PP-5, *n* = 10); vehicle-treated Tg mice (Tg-v, *n* = 7); 5-mg/kg PSM-04-treated Tg mice (Tg-PP-5, *n* = 6); 10-mg/kg PSM-04-treated Tg mice (Tg-PP-10, *n* = 6); donepezil-treated Tg mice (Tg-DP, *n* = 5).

From the *in vitro* studies, PSM-04 reduced the neurotoxicity induced by L-glutamate or oligomeric Aβ_1-42_. PSM-04 also reduced the oxidative stress induced by H_2_O_2_. Here, we investigated whether PSM-04 is therapeutically effective in 5xFAD mice. Currently, a biomarker for AD is unknown (Blennow et al., 2015). Thus, patients with AD usually visit the hospital when they have mild cognitive impairment. In 5xFAD mice, memory deficits are detected from 4–6 months of age (Zeng et al., 2015). Therefore, we administered PSM-04 4 months-old 5xFAD mice. We treated 5xFAD mice with 5 or 10 mg/kg of PSM-04 or 2 mg/kg of donepezil (dissolved in 0.3% CMC) by oral administration daily for 2 months. To investigate the alleviation of cognitive impairment, we performed a novel objective recognition (NOR) task. As shown in the schematic diagram of the NOR task (Figures 3B,C), we checked the time spent in object recognition; WT-v showed significantly longer time spent exploring the unfamiliar object (familiar, 79.85 ± 10.08 s; unfamiliar, 183.62 ± 26.02 s, *p* < 0.01), whereas Tg-v showed comparable time spent exploring familiar and unfamiliar objects (familiar, 126.54 ± 16.07 s; unfamiliar, 161.96 ± 37.85 s) (Figure 3H). The Tg mice treated with 5-mg/kg or 10-mg/kg PSM-04 exhibited significantly longer time spent exploring the unfamiliar object (5-mg/kg PSM-04: familiar, 70.9 ± 19.34 s; unfamiliar, 174.43 ± 66.68 s, and 10 mg/kg PSM-04 familiar, 68.99 ± 9.86 s; unfamiliar, 179.83 ± 16.27 s, *p* < 0.001) (Figure 3H). The memory index also increased in Tg mice treated with 5-mg/kg (familiar, 0.37 ± 0.07 s; unfamiliar, 0.63 ± 0.07 s, *p* < 0.05) or 10 mg/kg PSM-04 (familiar, 0.28 ± 0.03 s; unfamiliar, 0.72 ± 0.03 s, *p* < 0.001) compared with those treated with the vehicle (Figure 3I). There was no difference in total exploration time, speed, total distance, and frequency (Figures 3D–G). In this result, PSM-04 alleviated cognitive impairment in 5xFAD mice.

### PSM-04 Reduced Amyloid Plaques in the Hippocampus but Not in the Cortex

**FIGURE 4:**
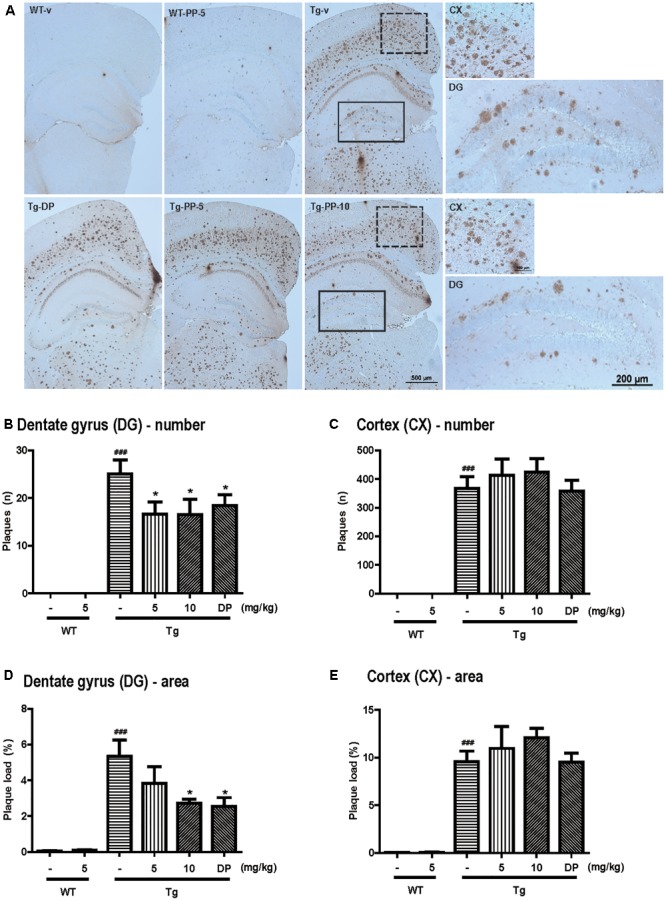
PSM-04 reduced amyloid plaques in the brains of 5xFAD mice. **(A)** Immunohistochemistry using a 6E10 antibody to stain amyloid plaques in the brains. Amyloid plaques were calculated as plaque counts **(B,C)** and plaque area **(D,E)** in the dentate gyrus **(B,D)** and the cortex **(C,E)**. In the dentate gyrus, amyloid plaque counts **(B)** and plaque area **(D)** significantly reduced in PSM-04-treated Tg brains compared with the non-treated Tg brains. However, there was no change in the plaque count **(C)** and plaque area **(E)** in the cortexes of PSM-04-treated Tg brains and non-treated Tg brains. The statistical analyses were performed by one-way ANOVA, and data are presented as the means ± SEM. ^###^ *p* < 0.001 vs. WT-v; ^∗^*p* < 0.05 vs. Tg-v. WT-v (*n* = 8); WT-PP-5 (*n* = 9); Tg-v (*n* = 7); Tg-PP-5 (*n* = 6); Tg-PP-10 (*n* = 6); and Tg-DP (*n* = 5).

Amyloid deposition is known to occur in 5xFAD mice at 1.5 months (Oakley et al., 2006). To investigate deposition of amyloid plaques in the hippocampus and cortex, immunohistochemistry was performed using 6E10 antibody (Figure 4A). Although WT mice showed no amyloid plaques, amyloid plaques were observed in Tg-vehicle mice in most regions of the brain (25 ± 3.02 n) (Figure 4B). In the dentate gyrus, the number of amyloid plaques was significantly reduced in the Tg-5-mg/kg (16.58 ± 2.58 n, *p* < 0.05) or Tg-10-mg/kg PSM-04-treated mice (16.50 ± 3.24 n, *p* < 0.05) (Figure 4B). Compared with Tg-vehicle mice (5.35 ± 0.91 n), amyloid plaque load was also reduced in the Tg-5-mg/kg (3.18 ± 0.89 n, *p* < 0.05) or Tg-10-mg/kg PSM-04-treated mice (2.73 ± 0.23 n, *p* < 0.05) in the dentate gyrus (Figure 4D). However, in the cortex, amyloid plaques were not significantly reduced in the Tg-5-mg/kg or Tg-10 mg/kg PSM-04-treated mice (Figures 4C,E).

These results indicated that PSM-04 reduced amyloid plaques in the dentate gyrus of the hippocampus but not of the cortex.

### PSM-04 Reduced Gliosis in the Dentate Gyrus

In 5xFAD mice, gliosis was detected at 6 months of age (Girard et al., 2014). To investigate whether gliosis was present in the dentate gyrus and it was reduced by PSM-04, we performed immunohistochemistry using GFAP antibody (Supplementary Figure S4). The number of GFAP-positive cells were increased in Tg mice (1.99 ± 0.26, *p* < 0.05) compared to WT mice (1 ± 0.10). Conversely, 5 mg/kg (1.06 ± 0.20, *p* < 0.05) or 10 mg/kg (1.01 ± 0.24, *p* < 0.05) of PSM-04 treatment reduced the number of GFAP-positive cells.

### PSM-04 Increased the Expression of SOD-2 and BDNF in the Brain of 5xFAD Mice

**FIGURE 5:**
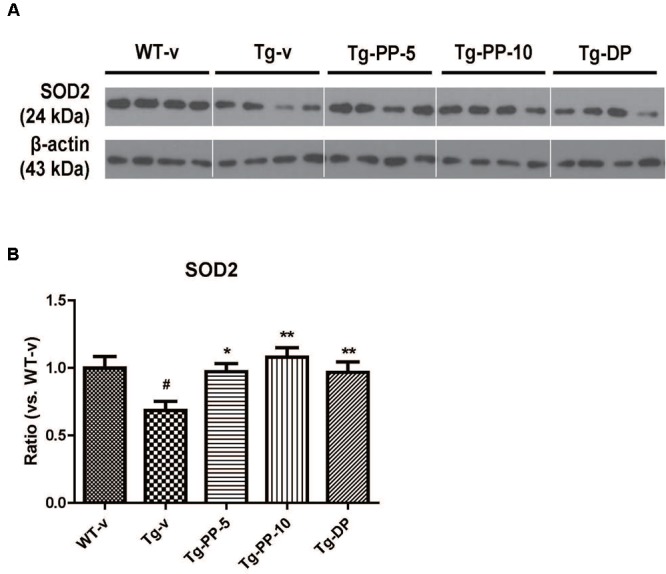
PSM-04 increased the expression of SOD-2 in the brain of 5xFAD mice. Representative Western blot demonstrating protein expression levels of SOD-2 in the hippocampus of each group. **(A)** Representative blot showing that SOD-2 protein levels increased in the hippocampal tissue lysates of PSM-04-treated Tg mice compared with those of the non-treated Tg mice. **(B)** The relative SOD-2 protein levels were shown as a ratio versus to the control (Tg-v). The statistical analyses were performed by one-way ANOVA, and data are presented as the means ± SEM. ^#^*p* < 0.05 vs. WT-v. ^∗^*p* < 0.01 and ^∗∗^*p* < 0.01 vs. Tg-v. WT-v (*n* = 8); WT-PP-5 (*n* = 9); Tg-v (*n* = 7); Tg-PP-5 (*n* = 6); Tg-PP-10 (*n* = 6); Tg-DP (*n* = 5).

Next, we tried to reveal the possible molecular mechanism under the neuroprotective effects of PSM-04 on AD. In a previous study, the overexpression of superoxide dismutase (SOD-2) reduced hippocampal superoxide and prevented memory impairment in 5xFAD mice (Massaad et al., 2009) and brain-derived neurotrophic factor (BDNF) signaling was shown to exert neuroprotective effects against Aβ peptide toxicity *in vivo* and *in vitro* (Arancibia et al., 2008). Therefore, the effect of PSM-04 treatment on the expression of SOD-2 and BDNF protein in the hippocampus of Tg mice was investigated. The expression levels of SOD-2 were decreased in Tg mice (*P* < 0.05) compared to WT mice and PSM-04 significantly increased SOD-2 protein level compared to Tg-vehicle mice (Figures 5A,B). In addition, mature.BDNF (mat.BDNF)/pro-BDNF ratio also was decreased in Tg mice (*P* < 0.05) compared to WT mice (Supplementary Figures S5A,B). While PSM-04 rescued SOD-2 protein level in Tg mice, PSM-04 slightly increased the mat.BDNF/proBDNF ratio, but not significant (Supplementary Figures 5A,B).

## Discussion

PSM-04, the root extract of *P. tenuifolia* Willdenow with improved bioabsorption, has been investigated for its neuroprotective effects in *in vitro* and *in vivo* models of Alzheimer’s disease. *In vitro* studies showed that PSM-04 dose-dependently reduced not only L-glutamate- or oligomeric Aβ-induced apoptosis but also H_2_O_2_-induced oxidative stress in primary cortical neurons. *In vivo* study suggested that the oral administration of PSM-04 prevented cognitive impairments and reduced amyloid plaques and gliosis in 5xFAD (Tg) mice brains. Furthermore, the administration of PSM-04 increased superoxide dismutase-2 (SOD-2) protein level in the hippocampus. It was concluded that PSM-04 could be considered for the treatment of neurodegenerative diseases, including AD.

In chronic neurodegenerative diseases, including Parkinson’s disease, Huntington disease, and AD, neuronal loss induced by neurotoxin is one of the important characteristics. Particularly, AD is characterized by the deposition of Aβ in the brain, which results in the neurotoxicity (Suh and Checler, 2002). In addition, oxidative stress is also an important factor in the pathogenesis of neurodegenerative diseases, including AD (Butterfield et al., 2001). Several studies have implicated oxidative stress in Aβ-induced neurotoxicity (Apelt et al., 2004; Mohmmad Abdul et al., 2006). Further, Aβ increases the levels of hydrogen peroxide and lipid peroxides (Behl et al., 1994). In addition, the intracellular accumulation of ROS may contribute to memory dysfunction and cognitive impairment in AD (Vitte et al., 2004). Therefore, Aβ induced neurotoxicity by oxidative stress, resulting in neuronal loss and cognitive dysfunction.

Several animal and human studies have reported that the extract of *P. tenuifolia* Willdenow reduces neurotoxicity and improves cognitive impairment (Park et al., 2002; Lee et al., 2009; Shin et al., 2009a; Liu et al., 2010). However, these studies used a “depression-like behavior” mice model or “scopolamine-induced amnesia” rat model, neither of which is an Alzheimer’s disease animal model.

In this study, we showed that PSM-04 itself did not induce cytotoxicity at concentrations of 0.1–5 μg/mL in primary cortical neurons but it rather reduced apoptosis from excessive L-glutamate-induced neurotoxicity (Supplementary Figure S2A). Because Aβ induces free radical oxidative stress and neurotoxicity in AD brain (Butterfield, 2002; Suh and Checler, 2002), we investigated whether PSM-04 has neuroprotective effects against oxidative stress induced by oligomeric Aβ in primary cortical neurons. We found that oligomeric Aβ-induced neurotoxicity was reduced by PSM-04 treatment in primary cortical neurons (Figure 1B) and that PSM-04 also reduced ROS generation induced by H_2_O_2_ (Figure 2). These results show that PSM-04 has neuroprotective effects against neurotoxicity induced by glutamate, oligomeric Aβ, or oxidative stress in primary cortical neurons. Next, we evaluated the *in vivo* therapeutic effects of PSM-04 in 5xFAD mice. At first, the NOR task was performed by the 6-month-old 5xFAD mice after 2-months of daily oral administration of PSM-04. The NOR test is a highly validated test for assessing recognition memory and is now among the most commonly used behavioral tests for mice (Leger et al., 2013). Tg-PSM-04 groups showed increased cognition of the novel object compared with the Tg-vehicle group. Regarding pathological changes, the Tg-PSM-04 mice showed reduced amyloid plaques and fewer GFAP-positive glial cells compared with Tg-vehicle mice. Next, we tried to determine the exact mechanism through which PSM-04 alleviated cognitive impairment and reduced amyloid plaques and gliosis in the dentate gyrus of the hippocampus region in the brain. Symptoms of AD in humans not only include memory loss but also neuropsychiatic, behavioral, and psychological symptoms of dementia (BPSD) (Lalonde et al., 2012). Although BPSD is very common in dementia, no pharmacological therapy or medication has yet been developed because of the lack of efficacy and safety (Huang et al., 2012). Recent researches show that BPSD occurs in the animal model of AD as well (Lalonde et al., 2012; Pfeffer et al., 2018) and is related with the dysfunction of NMDA neurotransmission (Huang et al., 2012) and Aβ-induced neurotoxicity (Tamano et al., 2016). Furthermore, studies on the antidepressant-like effect of *P. tenuifolia* Willdenow have been increasing in recent times (Shin et al., 2014; Zhu et al., 2017). Therefore, the neuroprotective effect of PSM-04 may alleviate BPSD in the 5xFAD mice; however, to establish this, the BPSD-like behavior in the 5xFAD mice needs to be further studied to investigate the possibility of PSM-04 treatment in BPSD.

We have looked at the proteins that can be involved with the neuroprotective effects of PSM-04, such as SOD and BDNF. SOD-2, known as manganese-dependent superoxide dismutase (MnSOD), is an enzyme that removes mitochondrial ROS and consequently prevents cell death (Pias et al., 2003). In AD, the interaction of ROS and AD is supported by clinical findings that revealed an upregulation of antioxidant enzymes, such as SOD-2 (De Leo et al., 1998). In addition, SOD-2 plays an anti-apoptotic role against oxidative stress (Becuwe et al., 2014). Further, the overexpression of SOD-2 protein prevents memory impairment in AD model mice (Massaad et al., 2009). In our result, SOD-2 protein levels increased in the hippocampal tissue lysates of PSM-04-treated Tg mice compared with the non-treated Tg mice. This result, including *in vitro* results, suggests that PSM-04 exerts neuroprotective effects by removing ROS and modulating the expression of ROS regulatory proteins such as SOD-2.

BDNF is a neurotrophin essential for long-term synaptic plasticity and memory formation as well as in synaptogenesis (Cunha et al., 2010). Dysregulation of BDNF signaling is involved in several neurodegenerative diseases, including AD (Schindowski et al., 2008). BDNF protein is formed from the cleavage of a 35-kDa proBDNF protein and is secreted as the 15-kDa mature BDNF (mat.BDNF) (Lessmann et al., 2003). Protease mediated conversion of proBDNF to mBDNF is considered to be an important mechanism contributing to activation-dependent synaptic competition in the central nervous system (CNS) (Lu et al., 2005). In our result, while mat.BDNF/pro-BDNF ratio was decreased in Tg mice, PSM-04 did not significantly rescue the mat.BDNF/proBDNF ratio (Supplementary Figure 5). These results suggest PSM-04 could relate with not processing mechanism of BDNF but another pathway.

Taken together, due to its ability to enhance neuroprotective effects by reducing apoptosis and ROS production, PSM-04 can be a potential pharmacological agent for treating neurodegenerative diseases, particularly in the treatment of Alzheimer’s disease.

## Author Contributions

K-AC and Y-HS supervised the project and designed the experiments. HP carried out the experiments, analyzed data, and wrote the manuscript. SK guided mouse behavior test. EN assisted in analyzing the data. All authors performed data quantification, discussed the results, and commented on the manuscript.

## Conflict of Interest Statement

The authors declare that the research was conducted in the absence of any commercial or financial relationships that could be construed as a potential conflict of interest.

## Supplementary Material

FIGURE S1 PSM-04 did not induce cytotoxicity and H_2_O_2_ treatment increased ROS production in primary cortical neurons. **(A)** Primary cortical neurons were treated with PSM-04 (0.1, 0.5, 1, and 5 μg/mL) for 12 h, and cell viability was then measured using the WST-1 assay. PSM-04 was not found to affect cytotoxicity in primary cortical neurons (*n* = 7∼10 wells per group, *N* = 5). **(B)** Primary cortical neurons were treated with various concentrations (50, 100, 200, or 400 μM) of H_2_O_2_ for 10 min. In the DCF-DA assay, fluorescence intensity was significantly increased in a dose-dependent manner, indicating that ROS production was increased by H_2_O_2_ treatment (*n* = 2 wells per group, *N* = 3). The statistical analyses were performed by *T*-test, and data are presented as the means ± SEM. ^∗^*p* < 0.05, ^∗∗^*p* < 0.01, and ^∗∗∗^*p* < 0.001 vs. control.

FIGURE S2 PSM-04 reduced cytotoxicity induced by L-glutamate in primary cortical neurons. The primary cortical neurons were treated with L-glutamate for 2 h, and then, cell viability was measured using the WST-1 assay and TUNEL staining. **(A)** Cell viability was the reduced by L-glutamate (L-glu) treatment in a dose-dependent manner (*n* = 4∼6 wells per group, *N* = 5). **(B–C)** The apoptotic cell death in primary cortical neurons treated with 100-μM L-glu for 2 h was visualized via TUNEL staining. **(B)** It was seen that 1-μg/mL PSM-04 or 200-ng/mL BDNF reduced the apoptotic cell death induced by 100-μM L-glu. **(C)** TUNEL-positive cells were counted and analyzed (*n* = 3 wells per group, *N* = 5). The statistical analyses were performed by *T*-test, and data are presented as the means ± SEM. ^#^*p* < 0.05 vs. control (NC). ^∗^*p* < 0.05, ^∗∗^*p* < 0.01, and ^∗∗∗^*p* < 0.001 vs. L-glutamate only.

FIGURE S3 PSM-04 reduced cytotoxicity induced by Aβ in primary cortical neurons. TUNEL staining was performed using the primary cortical neurons treated with PSM-04 for 12 h before being treated with 20-μM Aβ for 2 h. The apoptotic cell death was visualized in red fluorescence, and the nuclei were counterstained with DAPI.

FIGURE S4 PSM-04 reduced gliosis in the dentate gyrus. **(A)** Immunohistochemistry using a GFAP antibody to stain astrocyte in the cortex and dentate gyrus. **(B)** The number of GFAP-positive cells per area were counted in the dentate gyrus. The statistical analyses were performed by one-way ANOVA, and data are presented as the means ± SEM. ^#^*p* < 0.05 vs. WT-v; ^∗^*p* < 0.05 vs. Tg-v. WT-v (*n* = 8); WT-PP-5 (*n* = 9); Tg-v (*n* = 7); Tg-PP-5 (*n* = 6); Tg-PP-10 (*n* = 6); Tg-DP (*n* = 5).

FIGURE S5 PSM-04 slightly increased the ratio of mat.BDNF to proBDNF in the brain of 5xFAD mice. **(A)** Representative Western blot demonstrating protein expression levels of proBDNF and mat.BDNF in the hippocampus of each group. **(B)** The ratio of mat.BDNF to proBDNF normalized to the WT group. In the hippocampal lysates of PSM-04 treated Tg mice, the ratio of mat.BDNF / proBDNF slightly increased compared to the hippocampal lysates of Tg-v mice, but it was not significant. The statistical analyses were performed by one-way ANOVA, and data are presented as the means ± SEM. ^##^*p* < 0.01 vs. WT-v. WT-v (*n* = 8); WT-PP-5 (*n* = 9); Tg-v (*n* = 7); Tg-PP-5 (*n* = 6); Tg-PP-10 (*n* = 6); Tg-DP (*n* = 5).
