# Supplementary figures and images for: The Protective Effects of PSM-04 Against Beta Amyloid-Induced Neurotoxicity in Primary Cortical Neurons and an Animal Model of Alzheimer’s Disease
Source: Front Pharmacol. 2019 Jan 24;10:2. doi: 10.3389/fphar.2019.00002 (PMC6353859; doi:10.3389/fphar.2019.00002)

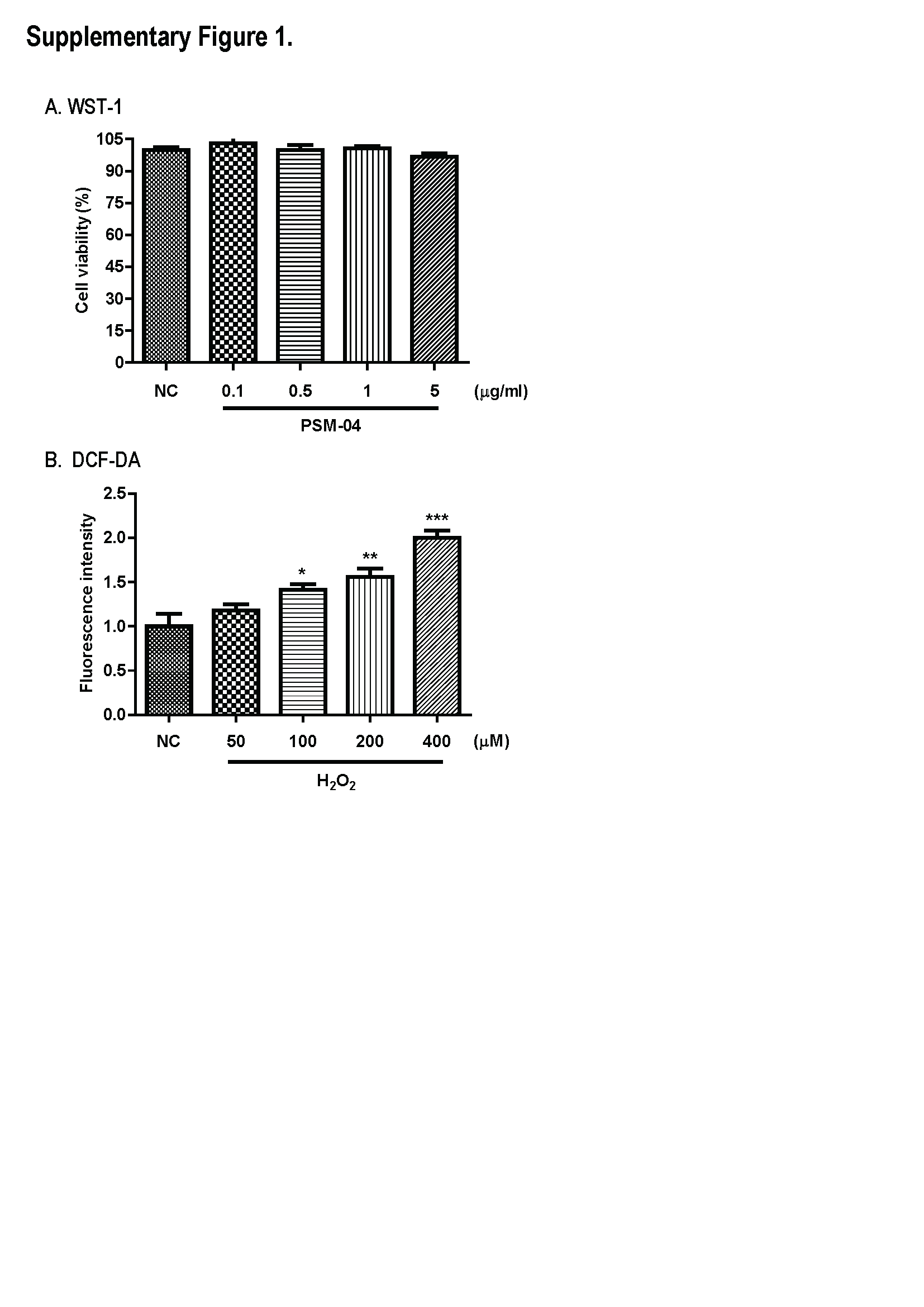

Supplement: FIGURE S1 — PSM-04 did not induce cytotoxicity and H2O2 treatment increased ROS production in primary cortical neurons. (A) Primary cortical neurons were treated with PSM-04 (0.1, 0.5, 1, and 5 μg/mL) for 12 h, and cell viability was then measured using the WST-1 assay. PSM-04 was not found to affect cytotoxicity in primary cortical neurons (n = 7∼10 wells per group, N = 5). (B) Primary cortical neurons were treated with various concentrations (50, 100, 200, or 400 μM) of H2O2 for 10 min. In the DCF-DA assay, fluorescence intensity was significantly increased in a dose-dependent manner, indicating that ROS production was increased by H2O2 treatment (n = 2 wells per group, N = 3). The statistical analyses were performed by T-test, and data are presented as the means ± SEM. ∗p < 0.05, ∗∗p < 0.01, and ∗∗∗p < 0.001 vs. control. [file Image_1.TIFF]

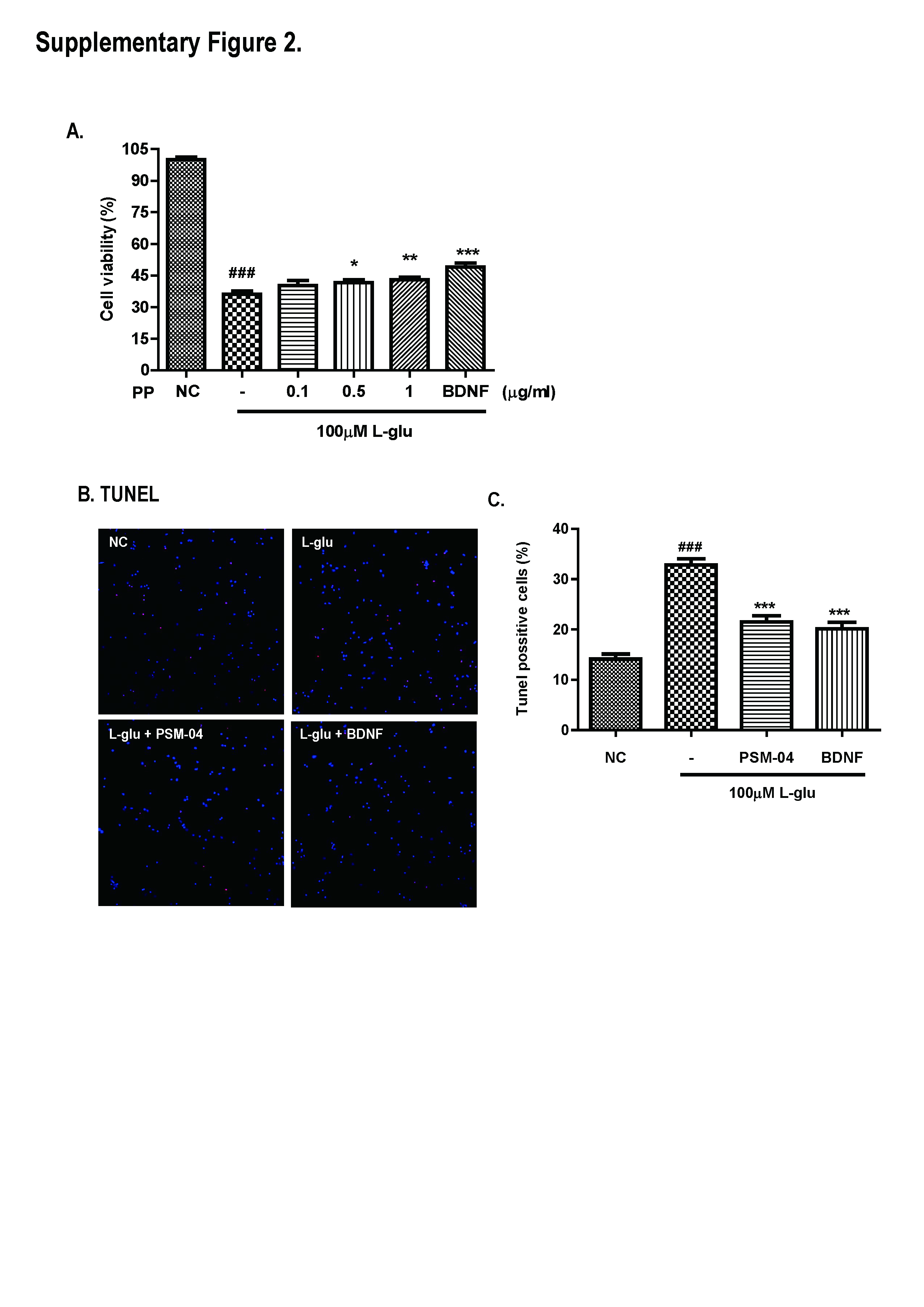

Supplement: FIGURE S2 — PSM-04 reduced cytotoxicity induced by L-glutamate in primary cortical neurons. The primary cortical neurons were treated with L-glutamate for 2 h, and then, cell viability was measured using the WST-1 assay and TUNEL staining. (A) Cell viability was the reduced by L-glutamate (L-glu) treatment in a dose-dependent manner (n = 4∼6 wells per group, N = 5). (B–C) The apoptotic cell death in primary cortical neurons treated with 100-μM L-glu for 2 h was visualized via TUNEL staining. (B) It was seen that 1-μg/mL PSM-04 or 200-ng/mL BDNF reduced the apoptotic cell death induced by 100-μM L-glu. (C) TUNEL-positive cells were counted and analyzed (n = 3 wells per group, N = 5). The statistical analyses were performed by T-test, and data are presented as the means ± SEM. #p < 0.05 vs. control (NC). ∗p < 0.05, ∗∗p < 0.01, and ∗∗∗p < 0.001 vs. L-glutamate only. [file Image_2.TIFF]

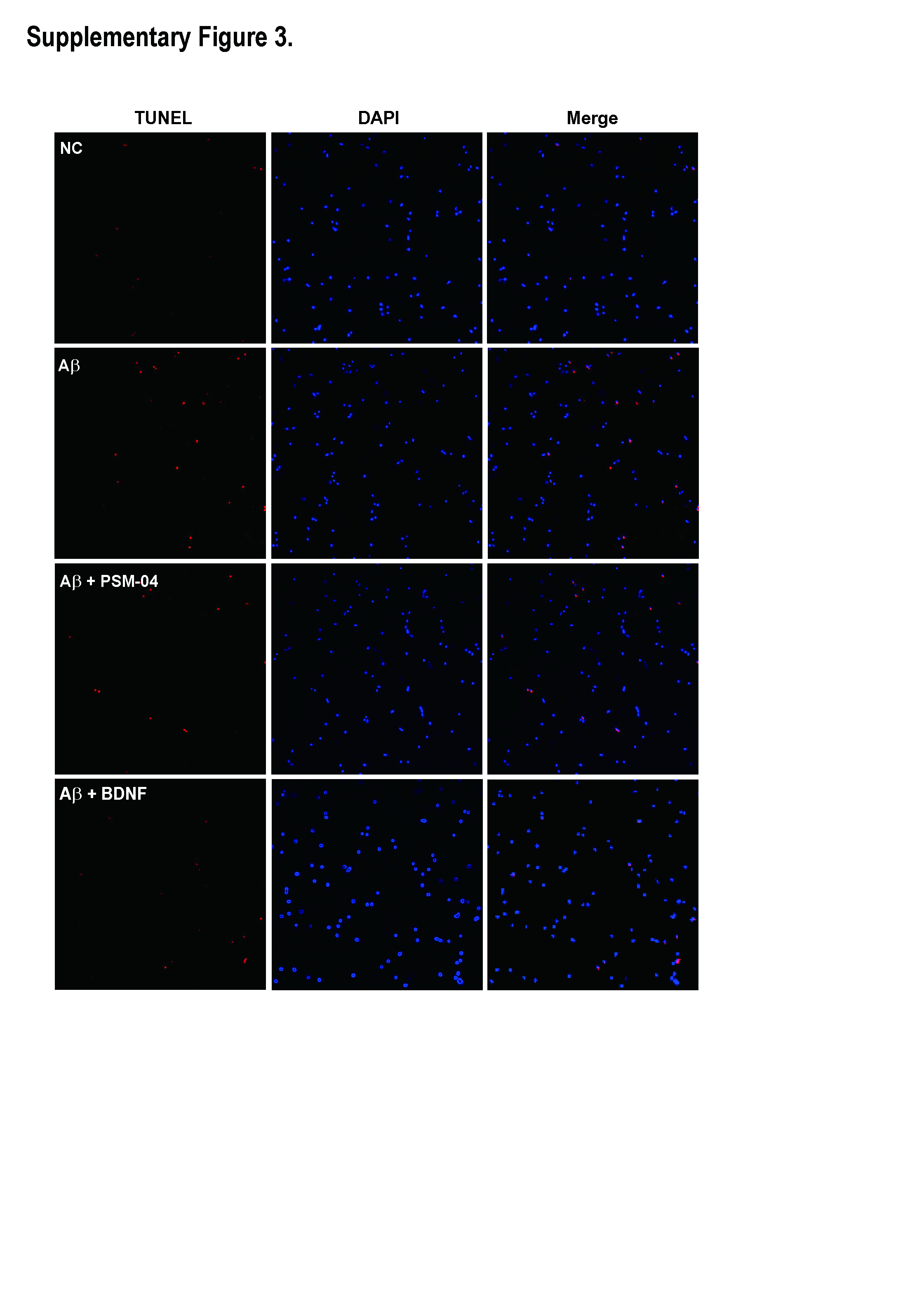

Supplement: FIGURE S3 — PSM-04 reduced cytotoxicity induced by Aβ in primary cortical neurons. TUNEL staining was performed using the primary cortical neurons treated with PSM-04 for 12 h before being treated with 20-μM Aβ for 2 h. The apoptotic cell death was visualized in red fluorescence, and the nuclei were counterstained with DAPI. [file Image_3.TIFF]

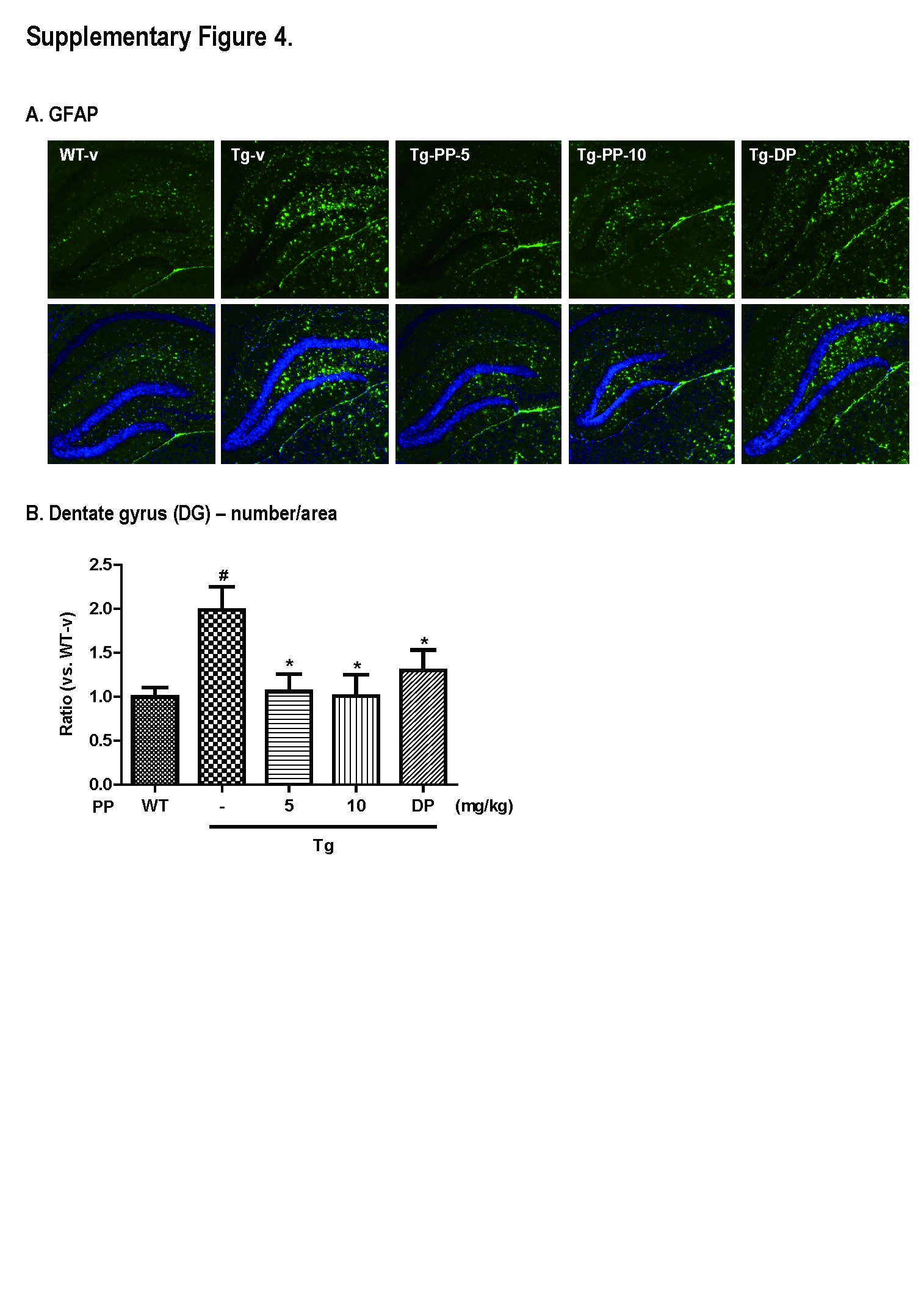

Supplement: FIGURE S4 — PSM-04 reduced gliosis in the dentate gyrus. (A) Immunohistochemistry using a GFAP antibody to stain astrocyte in the cortex and dentate gyrus. (B) The number of GFAP-positive cells per area were counted in the dentate gyrus. The statistical analyses were performed by one-way ANOVA, and data are presented as the means ± SEM. #p < 0.05 vs. WT-v; ∗p < 0.05 vs. Tg-v. WT-v (n = 8); WT-PP-5 (n = 9); Tg-v (n = 7); Tg-PP-5 (n = 6); Tg-PP-10 (n = 6); Tg-DP (n = 5). [file Image_4.TIFF]

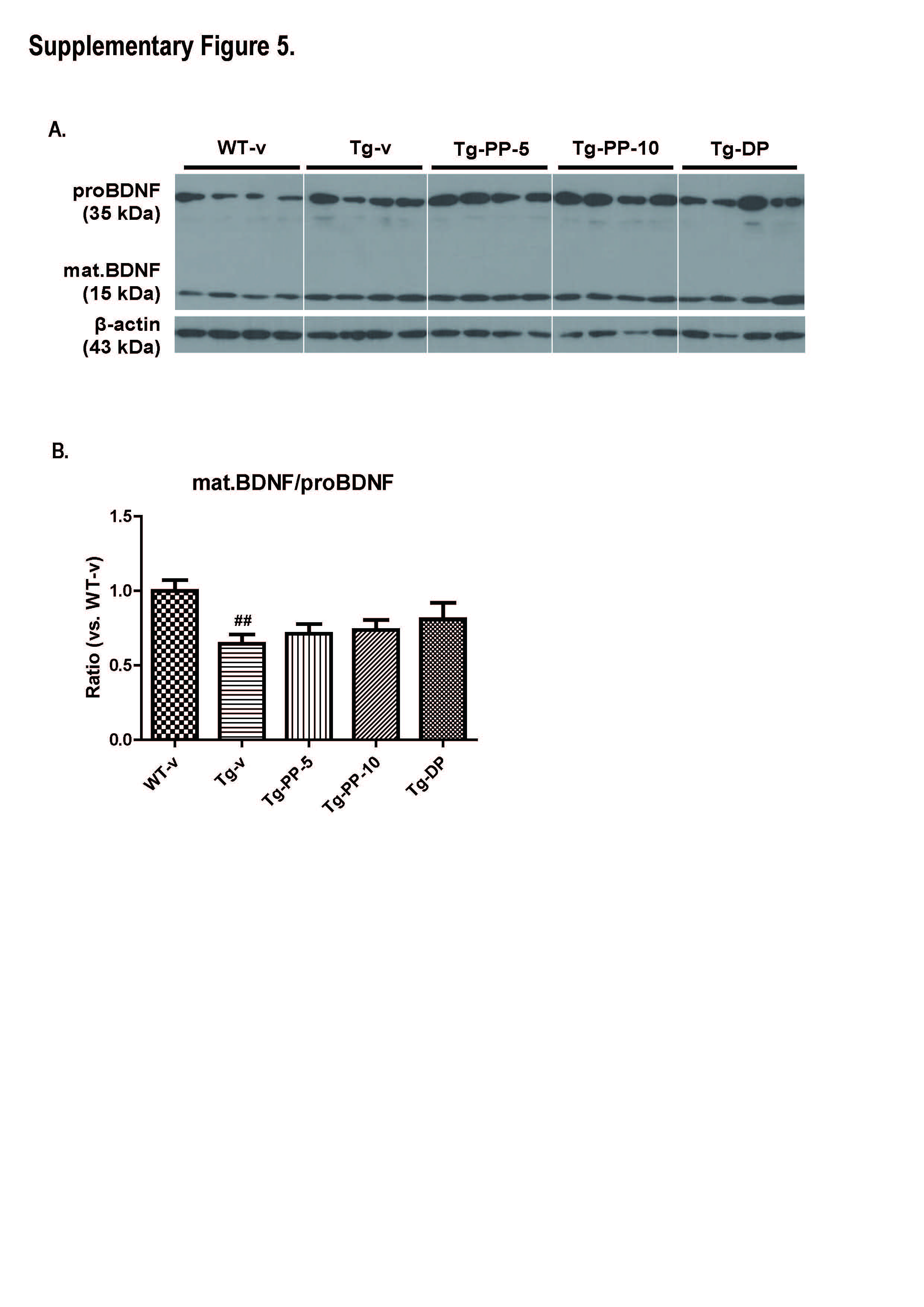

Supplement: FIGURE S5 — PSM-04 slightly increased the ratio of mat.BDNF to proBDNF in the brain of 5xFAD mice. (A) Representative Western blot demonstrating protein expression levels of proBDNF and mat.BDNF in the hippocampus of each group. (B) The ratio of mat.BDNF to proBDNF normalized to the WT group. In the hippocampal lysates of PSM-04 treated Tg mice, the ratio of mat.BDNF / proBDNF slightly increased compared to the hippocampal lysates of Tg-v mice, but it was not significant. The statistical analyses were performed by one-way ANOVA, and data are presented as the means ± SEM. ##p < 0.01 vs. WT-v. WT-v (n = 8); WT-PP-5 (n = 9); Tg-v (n = 7); Tg-PP-5 (n = 6); Tg-PP-10 (n = 6); Tg-DP (n = 5). [file Image_5.tiff]
